# LONG-TERM EFFICACY OF SPASTICITY-CORRECTIVE SURGERY AND BOTULINUM TOXIN INJECTIONS FOR UPPER LIMB SPASTICITY TREATMENT

**DOI:** 10.2340/jrm-cc.v8.42928

**Published:** 2025-05-11

**Authors:** Therese RAMSTRÖM, Johanna WANGDELL, Carina REINHOLDT, Trandur ULFARSSON, Lina Bunketorp KÄLL

**Affiliations:** 1 Institute of Clinical Sciences, Department of Hand Surgery at Sahlgrenska Academy, University of Gothenburg, Mölndal, Sweden; 2 Centre for Advanced Reconstruction of Extremities, Sahlgrenska University Hospital/Mölndal, Mölndal, Sweden; 3 Occupational and physiotherapy department, Sahlgrenska University Hospital/ Mölndal, Mölndal, Sweden; 4 Institute of Neuroscience and Physiology, Department of Clinical Neuroscience, Sahlgrenska Academy, University of Gothenburg, Gothenburg, Sweden; 5 Institute of Neuroscience and Physiology, Department of Health and Rehabilitation, Sahlgrenska Academy, University of Gothenburg, Gothenburg, Sweden

**Keywords:** botulinum toxin, muscle overactivity, outcome assessment, rehabilitation, tendon lengthening

## Abstract

**Objective:**

To evaluate the long-term efficacy of spasticity-corrective surgery and botulinum toxin treatment in patients with upper limb spasticity.

**Design:**

Pretest-posttest quasi-experimental study.

**Patients:**

Thirty-four patients with disabling spasticity.

**Methods:**

Patients were divided into 2 groups based on their treatment preference: the surgery group, which underwent tendon lengthening/release (*n* = 17), and the botulinum toxin injection group (*n* = 17). The primary outcome measure was the Modified Ashworth Scale. Secondary outcomes included range of motion, grip strength, and activity performance. Assessments were conducted at baseline for both groups, at 3 months following botulinum toxin injection, and at 6 months following surgery, with an additional peak-effect evaluation for botulinum toxin at week 5.

**Results:**

The surgery group demonstrated significantly greater reductions in composite Modified Ashworth Scale scores, with a mean change of 2.7 (SD 0.8), compared to the botulinum toxin group (1.1, SD 0.6 at peak; 0.3, SD 0.5 at long-term; *p* < 0.001). Surgery also led to significantly larger improvements in range of motion, grip strength, task performance, and patient satisfaction. While botulinum toxin effects were transient, surgery provided sustained benefits.

**Conclusion:**

Spasticity-corrective surgery achieves superior and longer-lasting benefits compared to botulinum toxin treatment in patients with disabling upper limb spasticity.

## LAY ABSTRACT

Spasticity is a condition where muscles contract uncontrollably, often occurring after injuries to the brain or spinal cord. This can cause complications like shortened muscles and difficulty performing daily tasks. This study evaluated the long-term effectiveness of 2 treatments for upper limb spasticity: surgery and botulinum toxin (BoNT) injections. Patients selected their preferred treatment, with 17 patients in each group. The primary outcome was on how much the spasticity improved, measured using the Modified Ashworth Scale (MAS). Secondary outcomes included range of motion and the ability to perform daily activities. Assessments were conducted before treatment, 3 months after BoNT injections, and 6 months after surgery, with an additional evaluation of BoNT’s peak effects at 5 weeks. The results showed that the surgery group experienced significantly greater improvements in MAS and secondary outcomes compared to the BoNT group. The benefits of surgery were long-lasting, while the effects of BoNT were temporary.

Spasticity, characterized by involuntary and sustained muscle contractions, is a common complication of stroke, traumatic brain injury (TBI), spinal cord injury (SCI), and other central nervous system (CNS) lesion-inducing conditions (1). A widely used definition of spasticity is a velocity-dependent increase in the tonic stretch reflex (muscle tone) with exaggerated tendon jerks” (2). However, this definition has been challenged, and others have proposed different descriptions in the medical literature; as such, no universally agreed definition is available today. Commonly reported figures estimate that spasticity affects 80% of patients with SCI (3), 60% of patients with TBI (4), and 30–40% of patients with stroke (5).

If left untreated, spasticity can lead to muscle shortening, contractures, joint deformities, pain, impairments in daily activities, and medical and psychological complications. However, in some patients, spasticity can be beneficial, aiding daily functions through key triggering strategies (6). Before planning an intervention, it is important to consider whether spasticity can be helpful in activity performance, or whether it is purely negative for the individual.

Various treatment options exist to manage spasticity, including *nonpharmacologic treatments* (stretching, muscle strength training, electrical stimulation) (7)*, pharmacologic treatments* (oral medication and intramuscular injections with botulinum toxin (BoNT) (8) and *surgical interventions* (intrathecal pumps, orthopaedic, and neurosurgery) (9). In many countries, BoNT injections are considered the gold standard of treatment for focal spasticity (10). To optimize the effect of surgical interventions and BoNT injections, treatment is commonly combined with a specific individualized rehabilitation program (adjunct therapies), such as passive and active range of motion (AROM) exercises, splinting, and strengthening exercises, training in activity, and training to correct faulty movement patterns (11).

BoNT injection has been proven to be safe and has been shown to reduce muscle tone and pain and improve passive functions. However, the effect of BoNT injections on active voluntary muscle function remains unclear (12). The surgical procedure used in the present study has been proven to be safe, with negligible complications and has been shown to reduce muscle tone and improve passive function, such as hand hygiene and caregiver burden, and active functions such as grasp ability (13). However, studies evaluating the effectiveness of alternative treatment approaches for adult patients with upper limb (UL) spasticity following CNS injuries in comparison to gold-standard methods, such as BoNT, are lacking. In 1 recent review (14), the authors stated the need for clinical trials comparing surgery with BoNT. Therefore, this study was designed with the primary aim of evaluating the long-term efficacy of spasticity-corrective surgery vs BoNT in patients with disabling UL spasticity. As a secondary aim, we compared the peak effects of the 2 treatments, hypothesizing that surgery would be more beneficial to patients than BoNT in the long term, with respect to function and activity performance (defined as the assessment at 6 months post-surgery and 3 months post BoNT injection). However, we further hypothesized that these methods would produce equivalent improvements when comparing the 6-month postsurgical outcome with the point of maximum benefit of BoNT (5 weeks post-injection).

## METHODS

### Study design and participants

This study used a pretest-posttest quasi-experimental design. Enrolment began in 2019 and was completed in June 2024. Owing to the Covid-19 pandemic, inclusion was halted between March 2020 and February 2022. The eligibility criteria for this study were as follows: (*i*) 18 years or above; (*ii*) problematic spasticity, characterized by a velocity-dependent increase in tonic stretch reflexes or intermittent or sustained involuntary muscle activity in the UL after stroke, TBI, or SCI; and (*iii*) patients treated at least 6 months after the injury event; and (*iv*) ongoing BoNT treatment in the UL, (*v*) a minimum of 3 months passed since the last BoNT injection: (*vi*). At least two muscles in the hand and wrist were considered for treatment (*vii*). For the BoNT group, a community occupational or physical therapist was assigned for post BoNT treatment; (*viii*) For the surgery group, medically stable to undergo surgery; (*ix*) No other severe UL injuries affecting the functional level.

**Fig. 1:**
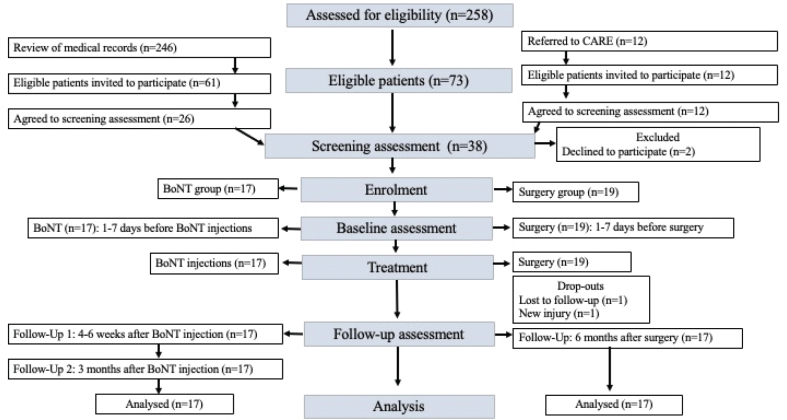
Flowchart of patient enrollment, treatment, and follow-up in botulinum toxin (BoNT) and surgery groups. (Explanation: This figure outlines the process of patient enrollment, treatment, and follow-up for individuals assigned to either the Botulinum Toxin (BoNT) group or the Surgery group. The flowchart provides a step-by-step overview, starting from the initial review of medical records to the final analysis of results. CARE: Center for advanced reconstruction of extremities; BoNT: Botulinum toxin; *n* = number).

Study participants were recruited using 2 parallel procedures: (*i*) Review of a hospital-based register of patients who had been treated or referred to the tonus clinic identified eligible patients, who were then sent information about the study, along with their contact information to the researcher responsible for the study. (*ii*) Patients with ongoing BoNT treatment who had been referred to the Center for Advanced Reconstruction of Extremities (C.A.R.E.), and were eligible for the present study were informed about the study and enrolment procedure. All presumptive study participants underwent a screening procedure by a primary examiner, to assess whether they met the study’s eligibility criteria. Further information regarding the study was provided at this point, with the opportunity to ask questions. Written informed consent was obtained if the patient met the inclusion criteria and consented to participate. All patients were offered either of the 2 treatment methods, while the treatment allocation was based on individual preferences. The flowchart of this study is presented in Fig. 1. All procedures were performed in compliance with relevant laws and institutional guidelines and were approved by the Regional Ethical Review Board in Gothenburg (No. 999-18) on 17 December 2018. The study was conducted in accordance with relevant ethical guidelines (Declaration of Helsinki).

### Interventions

*Spasticity-corrective surgery.* Surgical treatment was conducted as part of the routine care at C.A.R.E, Sahlgrenska University Hospital/Mölndal. Surgery included lengthening or releasing a tendon from its insertion, resulting in the consequent relaxation of the entire muscle-tendon unit; hence, the degree of muscle tension is reduced. The spasticity correction procedures were performed using a stair-step incision technique, followed by reattachment in the lengthened position using a side-to-side cross-stitch technique (15). The degree of tendon lengthening was decided by aiming for a normal resting length, which was estimated at full relaxation during general anaesthesia and with the remembrance of the preoperative status; 2–3 cm was usually sufficient. The day after surgery, wrapping and custom-made splints were fashioned to facilitate prolonged soft tissue stretching and prevent postoperative oedema. When possible, AROM exercises of the antagonist muscles of the lengthened muscles were performed on the first day after surgery, as was passive or AROM activation of the treated muscles. AROM exercises were allowed to achieve the maximum ROM, with no restrictions on the lengthened muscles or their antagonists. All patients returned to the ward 3 weeks after surgery for follow-up and inpatient rehabilitation of varying lengths, depending on the treatment regimen. Three weeks after surgery, splints were only used at night until 3 months after surgery, and were readjusted if needed. The continued training was individually tailored to meet each patient’s goals. The treatment concept used in this study has been previously described in detail (13, 16).

*Botulinum toxin treatment.* BoNT treatment was administered as part of routine care at the Tonus Clinic, Rehabilitation Medicine, Sahlgrenska University Hospital/Högsbo. All patients had previously received BoNT injections, and only 1 injection cycle was used in this study. All injections were performed by an experienced physician with a special interest in spasticity. Electrical stimulation (ES) was used for muscle localization using a hollow insulated monopolar needle connected to a portable ES machine. The approximate location of the target muscle was determined using standard anatomic landmarks. Once the needle electrode was positioned in the target muscle, ES was delivered through the needle electrode to the muscle to produce muscle contraction. The visual feedback of appropriate muscle contraction confirmed that the needle electrode was likely in the target muscle, and the appropriate botulinum BoNT dose was injected. For some of the patients, ultrasound (US) guidance was used for musculoskeletal procedural guidance in addition to ES. BoNT blocks certain chemical signals from the nerves, resulting in temporary relaxation of muscles. Normally, the effect wears off after 3 months, and its maximum (peak) effect typically occurs 4–6 weeks after injection (10). In the present study, the dose and number of injected muscles varied depending on the degree and extent of spasticity. The post-injection treatment protocol varied but commonly involved stretching, strength training of the antagonists, and, in some cases, splinting.

*Effects and functional implications of spastic muscles.* Spasticity in muscles can profoundly affect posture, breathing, and upper-body function. Spastic pectoral muscles promote shoulder internal rotation and adduction, leading to poor posture, restricted breathing, and challenges with dressing and washing the upper body.

Spasticity in the elbow flexors (biceps brachii, brachioradialis, and brachialis) keeps the elbow flexed and the forearm supinated (due to the biceps brachii), restricting reach and grasp. This can impair balance and walking and interfere with hygiene and dressing of the upper body.

The pronator teres, when spastic, forces the arm into a pronated position, making object grasping difficult and leading to compensatory shoulder movements during hand use.

Spastic wrist flexors (flexor carpi radialis, flexor carpi ulnaris, and palmaris longus) hold the wrist in a flexed position, making grasping difficult and sometimes interfering with dressing.

Spastic finger flexors (flexor digitorum superficialis and flexor digitorum profundus) cause the fingers to remain clenched, making it difficult to open the hand, release objects, maintain hand hygiene, or cut fingernails.

Thumb flexor spasticity pulls the thumb into the palm, preventing key pinch and making it difficult to hold or place objects in the hand. An overactive thumb abductor positions the thumb too close to the index finger, restricting cylindrical grasp and pinch. Additionally, spastic intrinsic muscles keep the metacarpophalangeal (MCP) joints flexed, further restricting hand opening.

**Fig. 2:**
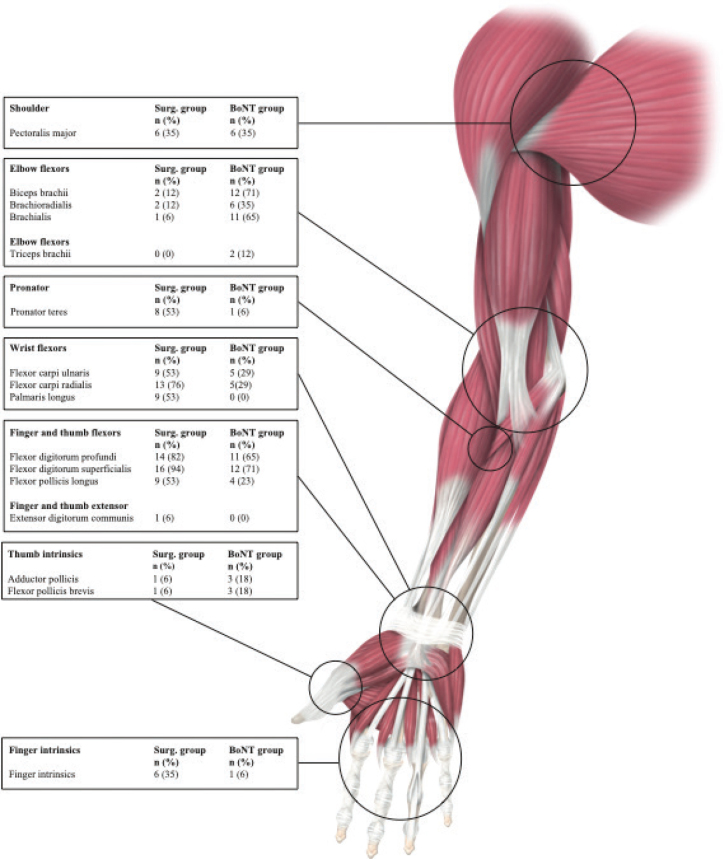
Distribution of target Muscles in surgery and Botulinum toxin treatment groups. (Explanation: The figure illustrates the distribution of muscle involvement between the 2 groups, Surgery and Botulinum Toxin (BoNT). The numbers indicate the count (n) and percentage (%) of patients in each group whose specific muscles were treated. Each group consisted of 17 patients. Abbreviation: Surg group: surgery group; BoNT group: Botulinum Toxin group; n: number).

The treated muscles of the 2 groups are presented in Fig. 2. Table II presents the target muscles and the objectives with the treatment, case by case, and Table III presents the effects and functional implications of spastic muscles.

### Outcome measures

The baseline data included demographics, clinical characteristics, and study-specific measurements. Owing to the differing time-course effects of BoNT and surgery, the assessment time points differed between the groups. For the primary research question (long-term effectiveness), the primary time point for the surgery group was the assessment at 6 months post-surgery and 3 months post-injection for the BoNT group, with an additional assessment at 1–5 weeks post-injection to capture the peak effects. The collected data included treated muscles, side effects, medication changes, and events from baseline to follow-up that could have affected the results. Participants also rated their satisfaction and perceived exertion with the treatment on a visual analogue scale (VAS) at the long-term follow-up.

All measures included in this study were based on the International Classification of Functioning, Disability, and Health (ICF) constructs (17). The *primary outcome measure* was muscle tone, measured using the Modified Ashworth Scale (MAS). The single-item MAS was measured on a 6-point scale from 0 (no increase in muscle tone) to 4 (affected part rigid in flexion or extension), with an additional point allocated at 1+ (slight increase in muscle tone). As such, the MAS provides a single score to represent spasticity in a specific movement. For analysis, the MAS scores of the treated muscles were summed to obtain a “composite spasticity score” for each participant.

### Secondary outcomes included measures within the following ICF domains

*Body function.* Self-rated pain intensity and experienced UL spasticity were measured using a VAS (18). AROM and passive range of motion (PROM) in the target joints were measured using a handheld goniometer in a sitting position, following a standard procedure (19). The capacity to achieve passive and active hand opening was rated using the following scale: hand closed, ¼ open, ½ open, ¾ open, or fully open. For analysis, the scale was scored from 0 (hand-closed) to –5 (fully open). The same grading system was applied to describe the resting position of the hands (20). Maximum handgrip strength was measured with a hydraulic hand dynamometer (JAMAR^®^ 5030J1, Sammons Preston Rolyan, USA) (21). Maximum pinch grip strength was measured using a Preston Pinch Gauge (European Bissel Healthcare Ltd., Winchester, England) (21).

*Activity.* The ability to grasp, move, and release objects was measured using the Grasp and Release Test (GRT) (22). The ability to actively and/or passively open one’s hand and actively grasp and release a cylindrical object was measured using a Cylinder Test. The test was divided into 5 subtests; (*i*) active cylinder grip-*normal*, (*ii*) active cylinder grip-*adapted*, (*iii*) active cylinder grip *self-assisted*, (*iv*) passive cylinder grip-*self-assisted*, and (*v*) passive cylinder grip-*examiner-assisted*. Self-rated arm and hand function (usefulness) were measured using the VAS (18). The Arm-Activity Measure (ArmA) questionnaire was used to capture difficulties in passive and active real-life arm functions (23).

*Participation.* Limitations in the prioritized daily activities were measured using the Canadian Occupational Performance Measure (COPM). The patients’ health-related quality of life (HRQoL) was assessed using the EuroQol 5-dimension questionnaire (EQ5D 5 L) (24).

### Sample size calculation

The calculation of sample size was based on the *a priori* defined difference to be detected, with an alpha level of 5% and a power goal of 80%, as well as the primary outcome variable MAS and previous findings (25, 26). The estimated changes in MAS-score in the surgery and BoNT groups were –1.3 standard deviation (SD) (0.7) and –0.6 (0.5), respectively. Based on this estimate, for the results to satisfy a power criterion of 80%, at least 14 participants were required in each of the 2 groups. We expected a dropout rate of 15%, and therefore aimed to include 17 individuals in each group to achieve 80% power.

### Statistical analyses

The demographic and baseline characteristics of the study participants were summarized using descriptive statistics. Due to the small number of participants, nonparametric tests were used. Significant analyses were conducted for outcome measures with 10 or more data points. Between-group differences in treatment efficacy were analysed by comparing pretest-posttest changes. The Mann-Whitney *U*-test was used for all comparisons. The main effect size was calculated using the equation *r* = Z/√N. For interpretation, the following guidelines were applied: an effect size *r* less than 0.3 indicates a small effect; *r* between 0.3 and 0.5 indicates a medium effect; and *r* greater than 0.5 indicates a large effect. The Wilcoxon signed-rank test was used for within-group treatment efficacy analyses. The statistical significance was set at 5%. The Holm-Bonferroni method was applied to reduce the possibility of Type I errors resulting from testing multiple hypotheses. To enable a comparison with previously reported findings, the results are presented using both mean values and medians.

## RESULTS

Seventeen patients in each group were included in the final analysis; the study flow is presented in Fig. 1.

The mean ages of the surgery and BoNT groups were 60 (26–76) and 55 (24–81) years, respectively, while the male: female ratios were 12:5 and 9:8, respectively. The detailed clinical characteristics of the 2 groups are presented in Table I.

**Table I:** [Demographic and clinical characteristics of the study cohort stratified by treatment groups]

Characteristics	Surgery group	BoNT group
**Patients**	17	17
**Age mean** (min-max)	60 (26–76)	55 (24–81)
**Sex**		
Male	12 (71)	9 (53)
Female	5 (29)	8 (47)
**Diagnosis**		
SCI	8 (47)	1 (6)
Stroke	8 (47)	14 (82)
TBI	1 (6)	2 (12)
**Time** (years) between injury/event and treatment mean (min-max)	7.5 (2–17)	11.8 (1–33)
**Ambulatory**		
Wheelchair	6 (35)	3 (18)
Wheelchair partial	4 (23)	5 (29)
Walking	7 (41)	9 (53)
**Regimen**		
HFR	5 (29)	6 (35)
LFR	7 (41)	5 (29)
NFR	5 (29)	6 (35)
**Functional score**		
1	4 (23)	7 (41)
2	2 (12)	2 ((12)
3	11 (65)	8 (47)
4	0 (0)	0 (0)
**Operated arm**		
Right	7 (41)	10 (59)
Left	10 (59)	7 (41)
**Number of complications**	3 (18)	1 (0.05)
**Adhered to the treatment regimen**	17 (100)	16 (94)

Data is reported as number (%) unless reported otherwise. Min: minimum; Max: maximum; n: numbers; SCI: spinal cord injuries; TBI: traumatic brain injuries; HFR: High-functioning regimen; LFR: Low-functioning regimen; NFR: Non-functioning regimen; BoNT: botulinum toxin.

**Table II A–B:** (A) Patient profiles, target muscles and treatment objectives in patients undergoing Botulinum injection treatment. (B) Patient profiles, target muscles and treatment objectives in patients undergoing surgery

Patient	Diagnosis	Sex	Regimen	Age	Target muscle	Product	Aim with treatment
**A**							
1	TBI	Female	LFR	40	BR(50U), Brachialis(50U) FDS(50U), FDP(60U)	Botox	Enable use as a supportive arm for daily activities, balance while walking
2	Stroke	Female	HFR	24	BR(25U), Brachialis(25U), FDS(25U), FDP(25U)	Botox	Grasping and releasing objects. Use of hand in daily activities and work.
3	Stroke	Male	LFR	55	Bic(150U), Brachialis(150U), FDS(150U), FDP (150U)	Dysport	Reduce pain due to increased tone, facilitate balance and walking with a straighter arm
4	Stroke	Male	NFR	68	Pec(100U), Bic(100U), BR(100U), Brachialis(50U), FCU(100U), FDP(100U)	Dysport	Facilitate hygiene; facilitate range of motion exercise to reduce risk of contractures
5	Stroke	Male	NFR	64	Pec(50U), Bic(50U), Brachialis(50U), FCU(75U), FCR(75U)	Xeomin	Facilitate hygiene, dressing
6	Stroke	Male	HFR	62	Bic(100U), BR(100U), Brachialis(100U), FDS(100U), ADP(40U)	Dysport	Grasping and releasing objects. Use of hand in daily activities, facilitate balance and walking with a straighter arm
7	Stroke	Female	HFR	39	FDS(140U), FDP(100U), FPL(60U)	Dysport	Use of hand in daily activities
8	Stroke	Female	LFR	62	Bic(180U), FDS(220U), FDP(220U), FPL(70U), FPB(50U), ADP(50U)	Dysport	Reduce pain, facilitate hygiene, enable use as a supportive arm for daily activities
9	Stroke	Male	HFR	54	Bic(25U), BR(25U), Brachialis(35U), Pron(20U), FDS(70U), FDP(25U)	Xeomin	Grip function, use of hand in daily activities
10	TBI	Female	NFR	67	Bic(100U), Brachialis(100U), FCU(100U), FDS(100U)	Dysport	Facilitate hygiene, dressing, improve range of motion to reduce contracture risk
11	Stroke	Male	NFR	81	Biceps(75U), BR(35U), Brachialis(40U), FCU(50U), FCR(50U), Intrinsic(25U), ADP(25U)	Botox	Facilitate hygiene, dressing, improve range of motion to reduce contracture risk
12	Stroke	Female	NFR	56	Pec(50U), Tric(50U), Bic(50U), Brachialis(25U), FDS(40U), FDP(25U), FPL(25U)	Botox	Facilitate hygiene, dressing, walking ability
13	Stroke	Male	LFR	66	Pec(50U), Bic(50U), Brachialis(50U), FCU(50U), FDP(50U), FPL(30U)	Botox	Balance while walking, enable use as a supportive arm for daily activities
14	Stroke	Male	HFR	52	Pec(40U), Tric(15U), Bic(15U), FDP(30U)	Botox	Reduce pain, use of hand in daily activities and leisure activities
15	Stroke	Female	LFR	42	FCR(75U), FDS(100U), FDP(125U),	Dysport	Enable use as a supportive arm for daily activities
16	Stroke	Female	NFR	42	FCR(60U), FDS(20U), FPB(10U)	Xeomin	Facilitate hygiene, dressing, improve range of motion to reduce contracture risk, cosmetic
17	SCI	Male	HFR	68	Pec(40U), Bic(50U), FCR(50U), FDS(20U), FPB(5U)	Xeomin	Grip function, use of hand in daily activities

Patient	Diagnosis	Sex	Regimen	Age	Target muscle	Aim with treatment	

**B**							
1	Stroke	Male	LFR	62	Pron, FCU, FCR, Intrinsic	Pain due to increase tone, support arm in daily life	
2	Stroke	Male	LFR	68	Pec, Pron, FCU, FCR, PL, FDS, FDP, Intrinsic	Enable use as a supportive arm for daily activities	
3	Stroke	Male	NFR	75	FDS, FDP, Intrinsic, FPL	Facilitate hygiene	
4	Stroke	Male	LFR	62	Pec, FCU, FCR, PL, FDS, FDP, ADP	Facilitate hygiene, support arm in daily activities	
5	Stroke	Male	NFR	74	Pec, Pron, FCU, FCR, PL, FDS, FDP, EDC	Facilitate hygiene	
6	Stroke	Female	LFR	35	Pron, FCR, PL, FDS, FPL	Balance while walking, enable use as a supportive arm for daily activities	
7	SCI	Female	LFR	56	Pec, Pron, FDS, FDP	Facilitate hygiene, facilitate dressing and posture in sitting, support hand	
8	SCI	Male	HFR	52	FDS, FDP, FPL	Grip function, use of hand in daily activities and leisure activities	
9	Stroke	Male	NFR	58	Pec, Bic, BR, Brachialis, FCU, FCR, FDS, FDP, FPL	Facilitate hygiene	
10	SCI	Male	HFR	59	Pron, FCU, FCR, FDS, FDP, Intrinsic,	Grip function, use of hand in daily activities and leisure activities	
11	SCI	Male	HFR	68	Pec, FCU, FCR, PL, FDS, FDP, FPL	Grip function, use of hand in daily activities	
12	SCI	Female	LFR	63	FCU, FCR, PL, FDS, FDP	Facilitate hygiene, enable use as a supportive arm for daily activities	
13	TBI	Male	NFR	45	Bic, Pron, FCR, FDS, FDP, Intrinsic, FPL	Facilitate hygiene	
14	Stroke	Female	NFR	69	FCU, PL, FDS, FDP, FPL, FPB	Facilitate hygiene, dressing,	
15	SCI	Male	LFR	76	FCR, PL, FDS, FDP, Intrinsic,	Grip function, use of hand in daily activities	
16	SCI	Male	HFR	26	BR, Pron, FCR, PL, FDS, FPD, FPL	Grip function, use of hand in daily activities and leisure activities	
17	SCI	Female	HFR	72	FCR, FDS, FPL	Grip function, use of hand in daily activities	

Abbreviation: U: units; TBI: Traumatic Brain Injuries; SCI: spinal cord injuries; HFR: High-functioning regimen; LFR; Low-functioning regimen; NFR; Non-functioning regimen; Pec: pectoralis; Tric: Triceps brachii; Bic: Biceps brachii; BR: Brachioradialis; Pro: pronator teres; FCU: flexor carpi ulnaris; FCR: flexor carpi radialis; PL: palmaris longus; FDS: flexor digitorum superficialis; FDP: flexor digitorum profundus; FPB: flexor pollicis brevis; FPL: flexor pollicis longus; ADP: adductor pollicis; ABP: abductor pollicis; EDC: Extensor digitorum communis.

**Table III:** Effects and functional implications of spastic muscles

Pectoral Muscles	Spastic pectoral muscles promote shoulder internal rotation and adduction, leading to poor posture, restricted breathing, and challenges with dressing and washing the upper body.
Elbow Flexors	Spasticity in the elbow flexors (biceps brachii, brachioradialis, and brachialis) keep the elbow flexed and the forearm supinated (due to the biceps brachii), restricting reach and grasp. This can impair balance and walking and interfere with hygiene and dressing of the upper body.
Forearm Muscles	The pronator teres, when spastic, forces the arm into a pronated position, making object grasping difficult and leading to compensatory shoulder movements during hand use.
Wrist and Finger Flexors	Spastic wrist flexors (flexor carpi radialis, flexor carpi ulnaris, and palmaris longus) hold the wrist in a flexed position, making grasping difficult and sometimes interfering with dressing. Finger flexors (flexor digitorum superficialis and flexor digitorum profundus) cause the fingers to remain clenched, making it difficult to open the hand, release objects, maintain hand hygiene, or cut fingernails.
Thumb muscles	Thumb flexor spasticity pulls the thumb into the palm, preventing key pinch and making it difficult to hold or place objects in the hand. An overactive thumb abductor positions the thumb too close to the index finger, restricting cylindrical grasp and pinch. Additionally, spastic intrinsic muscles keep the metacarpophalangeal (MCP) joints flexed, further restricting hand opening.

Six participants (2 in surgery group, 4 in BoNT group) reported changes in their routine or health condition which may have influenced the study results. In the surgery group, 1 participant’s focus on rehabilitation was negatively impacted by a family member’s stroke, while another had a reduction in their baclofen dose. In the BoNT group, 1 participant who had previously combined BoNT with intensive rehabilitation did not do so this time, another had an intensive rehabilitation period, and 2 participants had less training due to the pandemic.

The most commonly treated muscles in the surgery group were the finger flexors, wrist flexors, thumb flexors, and pronator teres. In the BoNT group, the most commonly targeted muscles were the fingers, wrist, thumb, and elbow flexors.

### Primary outcome measure

Between-group analyses revealed that surgery produced significantly greater improvement in MAS scores from baseline to follow-up compared to the BoNT group at both the peak and 3-month assessments (*p* < 0.001). The effect sizes were *r* 0.78 and *r* 0.85 between the surgery and BoNT peak and long-term assessment, respectively, indicating large effects.

**Fig. 3:**
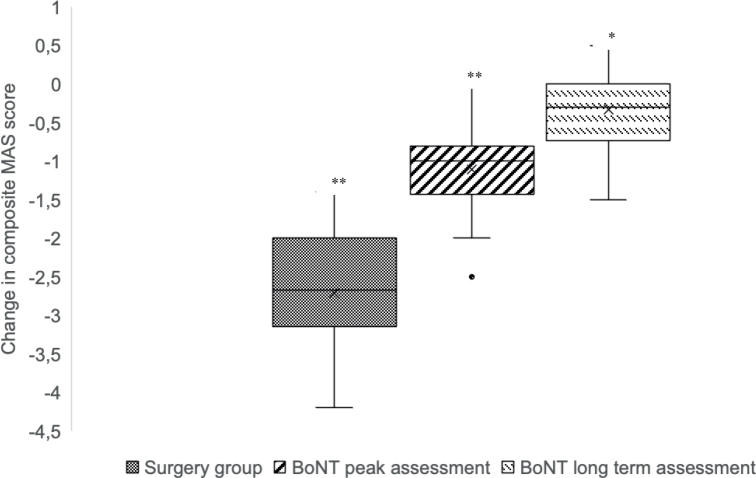
Comparison of mean differences in Modified Ashworth scale composite scores between surgery and Botulinum Toxin treatment groups. Explanation: This box plot illustrates the mean differences in MAS composite scores among 3 groups: the surgery group, BoNT peak assessment group, and BoNT long-term assessment group. The MAS score is a measure used to evaluate muscle spasticity, with lower scores indicating reduced spasticity. The box plot provides the median, interquartile range, and the overall range (whiskers) of the MAS scores for each group. The comparison highlights the differences in the effectiveness of the treatments over different periods, with the surgery group showing the most substantial reduction in MAS scores, followed by the BoNT peak and BoNT long-term assessments. *Indicates significantly difference below 0.005, **indicates significantly difference below 0.001*.* Abbreviation: MAS: modified Ashworth scale; BoNT: Botulinum toxin.

The within-group analyses demonstrated a significant reduction in the MAS score for the surgery group at 6 months, –2.7 (0.8), *p* < 0.001 and for the BoNT group at the peak assessment –1.1 (0.6), *p* < 0.001. In the long-term assessment, beneficial gains in MAS were still present in the BoNT group, yet diminished by –0.3 (0.5) *p* = 0.02. Fig. 3 summarizes the changes in the MAS scores.

### Secondary outcome measures

*Between treatment groups.* In the between-group analyses of secondary outcomes, the surgery group demonstrated significantly greater improvements than the BoNT group in 7 of the 17 secondary outcomes (peak assessment), including the resting position of the hand, pinch strength, perceived hand function, perceived spasticity, passive opening of the hand (subtest 4 of the cylinder test), and passive and active real-life arm function (Arma A and B). After applying the Holm-Bonferroni adjustment, 5 outcomes still showed significant improvement (Table IV). In the long-term assessment, 11 outcomes showed significant differences between the surgery and BoNT groups, with results in favour of the surgery group. After Holm-Bonferroni adjustment, 7 outcomes were still significantly improved: active opening of the hand, resting position of the hand, pinch strength, perceived spasticity, passive opening of the hand (subtest 4 of the cylinder test), and passive and active real-life arm function (Arma A and B, Table IV).

**Table IV:** Mean change in the secondary outcome measures, differences between the treatment groups

Outcome measure	*n*	Diff surgery. Baseline-long term. Mean(SD)/Median (min–max)	*n*	Diff BoNT Baseline -peak. Mean(SD)/Median (min–max)	*p*-value *r*-value	*n*	Diff BoNT Baseline -long term. Mean (SD)/Median (min–max)	*p*-value *r*-value
**Hand opening scale**								
Wrist neutral A	14	1.1 (0.8)	12	0.5 (0.7)	0.169	12	0.9 (0.3)	0.006*
		1.0 (0.0–2.0)		0.0 (0.0–2.0)	0.6346		0.0 (0.0–1.0)	0.3123
Wrist neutral P	17	0.8 (1.2)	17	0.4 (0.6)	0.375	17	0.0 (0.0)	**0.018**
		0.0 (0.0–4.0)		0.0 (0.0–2.0)	0.5409		0.0 (0.0–0.0)	0.1740
Wrist flexed A	14	0.6 (0.7)	12	0.7 (1.1)	0.539	12	0.4 (0.9)	0.722
		0.5 (0.0–2.0)		0.5 (–1.0–3.0)	0.1517		0.0 (0.0–3.0)	0.0908
Wrist flexed P	17	0.5 (0.9)	17	0.2 (0.4)	0.786	17	0.0 (0.3)	0.245
		0.0 (0.0–2.0)		0.0 (0.0–1.0)	0.0637		0.0 (–1.0–1.0)	0.3026
Resting position	17	1.7 (1.0)	17	0.5 (0.6)	< 0.001*	17	0.2 (0.6)	< 0.001*
		2.0 (0.0–4.0)		1.0 (–1.0–1.0)	0.6336		0.0 (–1.0–1.0)	0.7225
Grip strenght	14	–0.6 (4.3)	12	–2.3 (4.0)	0.347	12	0.9 (3.6)	0.297
		–0.5 (–7.4–8.0)		–1.4 (–11.4–3.7)	0.1867		0.1 (–4.2–6.0)	0.2070
Pinch strenght	14	0.8 (0.8)	13	–0.4 (1.1)	0.002*	13	–0.2 (1.1)	0.006*
		0.8 (–1.0–2.0)		–0.2 (–2.9–1.3)	0.5794		0.0 (–2.7–2.0)	0.5234
Pain(VAS)	17	–1.3 (2.7)	16	–1.1 (1.8)	0.790	16	0.2 (1.3)	0.179
		0.0 (–10.0–0.0)		0.0 (–5.0–2.0)	0.0534		0.0 (–2.0–3.0)	0.2703
Hand function (VAS)	17	2.3 (2.3)	16	0.7 (1.9)	**0.019**	16	0.6 (1.9)	**0.011**
		2.0 (0.0–9.0)		0.0 (–2.0–6.0)	0.4266		0.0 (–1.0–6.0)	0.4579
Cosmetic (VAS)	17	–0.3 (1.9)	16	–0.2 (2.1)	0.657	16	–0.1 (1.8)	0.471
		0.0 (–5.0–5.0)		0.0 (–3.0–4.0)	0.0893		0.0 (–4.0–5.0)	0.2418
Spasticity (VAS)	17	–4.7 (3.1)	16	–1.8 (1.6)	0.011*	16	–0.0 (1.3)	< 0.001*
		–4.0 (–10.0–0.0)		–2.1 (–4.0–2.0)	0.4416		0.0 (–3.5–2.0)	0.7582
GRT	10	27.4 (50.7)	8	18.1 (20.5)		8	14.4 (20.9)	
		19 (–7.0–174)		24.0 (–15.0–38.0)			10.0 (–9.0–51.0)	
**Cylinder Test**								
Subtest 1	12	29.2 (39.9)	8	10.0 (15.2)		8	1.2 (8.3)	
		10 (0–120)		0.0 (0.0–40.0)			0.0 (–10.0–20.0)	
Subtest 2	12	25 (26.4)	8	22.5 (19.1)		8	10.0 (15.2)	
		20 (0–80)		20.0 (0.0–50.0)			5.0 (–10.0–30.0)	
Subtest 3	12	27.5 (33.9)	9	30.0 (28.7)		9	15.5 (17.4)	
		15 (–10–100)		40.0 (0.0–80.0)			20.0 (0.0–50.0)	
Subtest 4	16	36.2 (36.8)	16	11.2 (15.9)	**0.043**	16	–6.9 (26.8)	< 0.001*
		35 (0–120)		10.0 (–10.0–40.0)	0.3659		0.0 (–70.0–20.0)	0.5297
Subtest 5	11	41.8 (31.6)	5	22.0 (19.2)		5	14.0 (20.7)	
		30 (0–90)		20.0 (0.0–50.0)			10.0 (–10–40.0)	
COPM-P	12	2.8 (1.1)	14	1.7 (1.4)	0.067	14	1.0 (1.6)	**0.011**
		3.0 (0.8–4.8)		1.6 (–0.6–5.0)	0.3588		0.1 (–0.6–4.0)	0.4910
COPM-S	12	2.9 (1.4)	12	2.7 (2.1)	0.887	12	1.6 (1.5)	**0.045**
		2.8 (–0.6–5.0)		2.7 (–0.2–5.4)	0.0292		1.5 (0.0–4.0)	0.4133
ArmA a	17	–11.1 (6.0)	17	–4.1 (4.6)	0.001*	17	–0.2 (4.8)	< 0.001*
		–12.0 (–21.0–0.0)		–4.0 (–14–3.0)	0.5385		0.0 (–6.0–13.00)	0.7316
ArmA b	17	–8.0 (7.3)	17	–2.5 (7.1)	**0.031**	17	–1.5 (4.3)	0.004*
		–6.0 (–20.0–0.0)		–1.0 (–18.0–16.0)	0.3712		0.0 (–12.0–9.0)	0.4925
Eq5dl VAS	17	7.8 (13.4)	16	2.0 (16.5)	0.363	16	9.0 (13.9)	0.929
		10.0 (–15.0–40.0)		0.0 (–40.0–30.0)	0.1636		3.0 (–10.0–35.0)	0.0189

Diff: difference; SD: standard deviation; Min: minimum; Max: maximum; n:numbers; BoNT: Botulinum Toxin Injection; A: active; B: passive; VAS: visual analogue scale; GRT: Grasp and Release Test; COPM: Canadian Occupational Performance Measure; P: performance scale; S: satisfaction scale; ArmA: Arm Activity Measure; ArmA a: passive subscale; ArmA b: active subscale. Statistical analyses of changes in median scores were made with The Mann-Whitney *U*-test for *n* ≤ 10, *p* < 0.05 were considered significant and are presented in bold numbers, significant values after the Holm- Bonferroni method was applied are presented with *. The main effect size was calculated using the equation *r* = Z/√N. For interpretation, the following guidelines were applied: an effect size *r* less than 0.3 indicates a small effect, *r* between 0.3 and 0.5 indicates a medium effect, and *r* greater than 0.5 indicates a large effect.

Following treatment completion, all patients were asked to rate their satisfaction with the results on a VAS, as well as how demanding they perceived the treatment. They were also asked as to whether they would recommend the treatment to someone else (yes or no). The mean satisfaction score was 7.4 (1.8) and 5.9 (2.6) in the surgery and BoNT groups, respectively. The mean score of how demanding they perceived the treatment was 3.5 (2.8) in the surgery group and 3.5 (3.2) in the BoNT group. Seventeen patients would recommend surgery to others, while 15 would endorse BoNT injections. However, 2 patients would not recommend BoNT treatment.

*Within the treatment group.* The findings of the secondary outcome measures are presented in Table VA–B. Within-group analyses demonstrated that the participants who underwent surgery showed significantly greater improvements in 21 of the 26 secondary outcomes. After applying the Holm-Bonferroni adjustment, 11 outcomes remained significantly improved. Within-group analyses revealed significant improvements in 10 out of 18 secondary outcomes in the BoNT group (peak assessment) and 3 out of 18 in the long-term assessment. After applying the Holm-Bonferroni adjustment, 5 outcomes remained significantly improved at the peak assessment and 2 at the long-term assessment.

**Table V A–B:** (A) Changes from baseline to the 6 months follow-up in the secondary outcome measures for the surgery group

Outcome measure	*n*	Baseline mean (SD)/median (range)	6 months mean (SD)/median (range)	Diff mean (SD)/median (range)	*p*
**Range of motion**					
Shoulder abduktion A	6	38.3 (34.3)	53.3 (36.1)	15 (18.7)	
		25 (0–90)	55 (0–90)	10 (0–50)	
Shoulder abduktion P	6	77.5 (12.5)	113.3 (20.6)	35.8 (22.4)	
		77.5 (60–90)	115 (90–140)	37.5 (0–70)	
Supination A	7	5.7 (28.3)	70.7 (28.6)	65.0 (25.8)	
		0 (–45–45)	80 (10–90)	80 (15–90)	
Supination P	9	58.3 (35)	86.1 (9.9)	27.8 (30.2)	
		80 (0–90)	90 (60–90)	10 (0–70)	
Wrist extension A	11	25.9 (26.4)	57.3 (21.4)	31.4 (14.1)	0.003*
		0 (0–9.5)	0 (0–9.5)	0 (–5–5)	
**Hand opening scale**					
Wrist neutral A	12	3.0 (0.8)	4.0 (0.7)	0.5 (0.7)	**0.006**
		3 (2–4)	4 (3–5)	0.0 (0.0–2.0)	
Wrist flexed A	12	4.0 (0.8)	4.6 (0.5)	1.0 (0.7)	**0.034**
		4 (2–5)	5 (245)	1.0 (0.0–2.0)	
Wrist neutral P	17	4.1 (1.2)	4.9 (0.2)	0.8 (1.2)	**0.011**
		4 (1–5)	5 (4–5)	0.0 (0.0–4.0)	
Wrist flexed P	17	4.5 (0.8)	5 (0.0)	0.5 (0.9)	**0.046**
		5 (3–5)	5 (5–5)	0.0 (0.0–2.0)	
Resting position	17	1.8 (0.7)	3.5 (0.8)	1.7 (1.0)	< 0.001*
		2 (1–3)	4 (2–5)	2.0 (0.0–4.0)	
Thumb-indexfinger	12	6.3 (4.5)	9.5 (4.2)	2.7 (4.5)	0.053
		7 (0–14)	10 (3–16)	2 (–3.5–13.5)	
First webspace	12	6.2 (3.5)	7.5 (3.0)	1.3 (3.7)	0.285
		6 (0–12)	7.2 (3–11)	0.7 (–5–8)	
GRT	10	79.1 (63.1)	109.2 (79.9)	27.4 (50.7)	**0.028**
		55.5 (19–190)	89 (16–231)	19 (–7–174)	
**Cylinder Test**					
Subtest 1	12	13.3 (31.4)	42.5 (48.1)	29.2 (39.9)	**0.027**
		31.4 (0–90)	25 (0–120)	10 (0–120)	
Subtest 2	12	31.7 (37.4)	56.7 (44.8)	25 (26.4)	**0.012**
		10 (0–100)	60 (0–130)	20 (0–80)	
Subtest 3	12	52.5 (47.3)	80 (47.3)	27.5 (33.9)	**0.015**
		65 (0–150)	80 (0–150)	15 (–10–100)	
Subtest 4	16	71.2 (53.5)	107.5 (54.7)	36.2 (36.8)	0.002*
		65 (0–150)	135 (0–150)	35 (0–120)	
Subtest 5	11	93.6 (54.7)	135.4 (54.7)	41.8 (31.6)	**0.008**
		120 (0–150)	120 (0–150)	30 (0–90)	
COPM-P	12	2.3 (1.0)	5.4 (1.1)	2.8 (1.1)	< 0.001*
		2.7 (1–4)	5.3 (3–7)	3 (0.8–4.8)	
COPM-S	12	2.2 (5.1)	5.1 (1.4)	2.9 (1.4)	< 0.001*
		2.1 (1–3)	5.6 (1–7)	2.8 (–0.6–5)	
ArmA subtest a	17	16.2 (4.6)	5.1 (3.5)	11.2 (6.0)	< 0.001*
		17 (10–23)	5 (0–13)	–12 (–21–0)	
ArmA subtest b	17	41.2 (10.3)	33.2 (15.4)	8.0 (7.3)	< 0.001*
		48 (21–51)	38 (4–50)	–6 (–20–0)	
Eq5dl	17	63.5 (23.0)	71.3 (20.9)	7.8 (13.4)	**0.028**
		70 (10–90)	75 (20–100)	10 (–15–40)	

Diff: difference; SD: standard deviation; Min: minimum; Max: maximum; n: numbers; FU1: follow-Up 1; FU2: Follow- Up 2; LT: long term; BoNT: Botulinum Toxin Injection; A: active; P: passive; VAS: visual analogue scale; GRT: Grasp and Release Test; COPM: Canadian Occupational Performance Measure; P: performance scale; S: satisfaction scale; ArmA: Arm Activity Measure; ArmA a: passive subscale; ArmA b: active subscale. Statistical analyses of changes in median scores were made with a Wilcoxon signed-rank test for *n* ≥ 10; *p* < 0.05 were considered significant and are presented in bold numbers, significant values after the Holm- Bonferroni method was applied are presented with *.

**Table V A–B:** (B) Changes from baseline to peak and lont-term follow-up in the secondary outcome measures for the BoNT group

Outcome measure	*n*	Baseline mean (SD) median (min–max)	FU1 (peak) mean (SD) median (min–max)	Mean diff mean (SD) median (min–max)	*p*	FU2 (LT) mean (SD) median (min–max)	Mean diff mean (SD) median (min-max)	*p*
**Range of motion**								
Shoulder abduktion P	6	93.3 (33.3)	113.3 (18.6)	20 (22.8)		96.7 (18.6)	3.3 (25.0)	
		100 (30–120)	120 (90–130)	15 (0–60)		95 (80–130)	25 (–20–50)	
Elbow extension A	8	16.2 (14.1)	9.4 (10.8)	–6.9 (11.0)		13.7 (10.3)	–2.5 (11.9)	
		15 (0–40)	5 (0–25)	–5 (–20–10)		15 (0–30)	0 (–20–10)	
Elbow extensionP	14	17.5 (25.8)	10.0 (19.3)	–7.5 (9.7)	**0.014**	14.6 (25.0)	–2.8 (6.1)	0.102
		10 (0–75)	0 (0–65)	–5 (–30–0)		0 (0–75)	0 (–10–10)	
Wrist extension P	8	26.2 (34.6)	42.5 (35.7)	16.2 (16.0)	**0.014**	28.7 (41.5)	2.5 (16.7)	
		15 (–10–90)	30.0 (0–90)	10 (0–40)		10 (–10–90)	0 (–20–40)	
**Hand opening scale**								
Wrist neutral A	10	2.2 (1.6)	2.8 (1.6)	0.5 (0.7)	**0.034**	2.3 (1.6)	0.1 (0.3)	0.317
		1 (1–5)	2.5 (1–5)	0 (0–2)		1.5 (1–5)	0 (0–5)	
Flexed wrist A	10	2.9 (1.7)	3.7 (1.6)	0.8 (1.1)	0.054	3.4 (1.6)	0.5 (1.0)	0.102
		2.5 (1–5)	4.5 (1–5)	1 (–1–3)		3.5 (1–5)	0 (0–3)	
Wrist neutral P	17	3.9 (1.0)	4.3 (0.9)	0.4 (0.6)	**0.020**	3.9 (1.0)	0.0 (0.0)	1.0
		4 (2–5)	5 (2–5)	0.0 (0.0–2.0)		4 (2–5)	0.0 (0.0–0.0)	
Flexed wrist P	17	4.5 (0.9)	4.8 (0.6)	0.2 (0.4)	**0.046**	4.5 (0.9)	0.0 (0.3)	1.0
		5 (2–5)	5 (3–5)	0.0 (0.0–1.0)		5 (2–5)	0.0 (–1.0–1.0)	
Resting position	17	2.0 (1.0)	2.5 (0.9)	0.5 (0.6)	0.011*	2.2 (1.0)	0.2 (0.6)	0.102
		1 (1–4)	3 (1–4)	1.0 (–1.0–1.0)		3 (1–4)	0.0 (–1.0–1.0)	
Thumb-indexfinger	8	5.25 (5.7)	5.2 (5.9)	0.0 (0.5)		6.3 (6.5)	1.1 (1.8)	
		4 (0–16)	3.5 (0–16)	0 (–1–1)		5.5 (0–17)	0.5 (–1–4)	
First webspace	8	4.4 (4.6)	4.6 (4.7)	0.2 (1.1)		4.9 (4.8)	0.5 (1.6)	
		4.4 (0–12)	4 (0–11)	0.5 (–1–4)		4 (0–12)	0 (–0.5–4.5)	
Grip strenght	12	15 (–10–90)	30.0 (0–90)	10 (0–40)	0.07	10 (–10–90)	0 (–20–40)	0.380
		8.8 (2–30)	6 (0.2–26)	–1.4 (–4.2–6)		10 (0.9–36)	0.1 (–4.3–6)	
Pinch strenght	13	3.5 (2.9)	3.1 (3.2)	0.4 (1.1)	0.195	3.3 (3.2)	0.2 (1.1)	0.476
		2.2 (0–10)	1.7 (0–10)	–0.2 (–2.9–1.3)		2 (0–6)	0 (–2.7–1.7)	
Pain	16	2.7 (2.9)	1.6 (1.7)	1.1 (1.8)	**0.035**	3.0 (2.6)	0.2 (1.3)	0.455
		2.7 (0–6)	1.5 (0–4)	0 (–5–2)		2.5 (0–7)	0 (–2–3)	
Hand function	16	1.7 (1.9)	2.4 (2.9)	0.7 (1.9)	0.180	2.3 (2.6)	0.6 (0.5)	0.206
		1.5 (0–6)	1.5 (0–9)	0 (–2–6)		1.5 (0–9)	0 (–1–6)	
Cosmetic	16	3.5 (3.2)	3.2 (3.3)	0.2 (2.1)	0.638	3.4 (3.5)	0.1 (0.5)	0.791
		3.5 (3.2)	3.2 (3.3)	0.2 (2.1)		3.4 (3.5)	0.1 (0.5)	
		3 (0–10)	2.5 (0–10)	0 (–3–4)		3 (0–10)	0 (–4–5)	
spasticity	16	7.5 (1.3)	5.7 (2.2)	1.8 (1.6)	< 0.001*	7.4 (1.7)	0.0 (1.3)	0.898
		7.6 (5–10)	5 (2–10)	–2.1 (–4–2)		7.5 (3–10)	0 (–3.5–2)	
GRT	8	52.8 (35.4)	71.0 (49.1)*	18.1 (20.5)		67.2 (52.7)	14.4 (20.9)	
		42.5 (16–130)	66 (15–167)	24 (–15–38)		52 (14–181)		
**Cylinder Test**								
Subtest 1	8	28.7 (47.0)	38.7 (57.7)	10 (15.2)		30 (53.2)	1.2 (8.3)	
		0 (0–130)	0 (0–150)	0 (0–40)		0 (0–150)	0 (–10–20)	
Subtest 2	8	48.7 (46.7)	71.2 (51.9)	22.5 (19.1)		58.7 (50.8)	10 (15.2)	
		55 (0–130)	85 (0–150)	20 (0–50)		55 (0–150)	5 (–10–30)	
Subtest 3	9	58.9 (59.5)	89.9 (60.1)	30 (28.7)		74.4 (56.4)	15.5 (17.4)	
		70 (0–150)	110 (0–150)	40 (0–80)		90 (0–150)	20 (0–50)	
Subtest 4	16	87.5 (46.1)	98.7 (45.6)	11.2 (15.9)	**0.015**	80.6 (51.8)	–6.9 (26.8)	0.641
		85 (0–150)	105 (0–150)	10 (–10–40)		85 (0–150)	0 (–70–20)	
Subtes 5	5	84.0 (46.7)	106.0 (36.5)	22 (19.2)		98.0 (50.7)	14 (20.7)	
		70.0 (30–150)	110.0 (50–150)	20 (0–50)		90 (40–150)	10 (–10–40)	
COPM-P	14	2.7 (1.4)	4.5 (2.2)	1.7 (1.4)	< 0.001*	3.8 (2.1)	1.0 (0.4)	**0.028**
		2 (1–6)	4.8 (1–9)	1.6 (–0.6–5.0)		3.5 (1–9)	0.1 (–0.6–4.0)	
COPM-S	12	2.1 (1.3)	4.8 (2.8)	2.7 (2.1)	< 0.001*	3.7 (2.4)	1.6 (1.5)	0.004*
		1.7 (1–5)	5.6 (1–9)	2.7 (–0.2–5.4)		3.4 (1–9)	1.5 (0.0–4.0)	
ArmA a	17	14.1 (7.0)	9.9 (5.9)	4.1 (4.6)	0.002*	13.9 (7.8)	0.2 (4.8)	0.882
		15 (1–23)	8 (1–24)	–4 (–14–3)		15 (1–30)	0.0 (–6–13)	
ArmA b	17	43.6 (10.5)	41.1 (12.6)	2.5 (7.1)	0.163	42.1 (12.0)	1.5 (4.3)	0.175
		49 (15–52)	48 (9–52)	–1 (–18–16)		48 (8–52)	0 (–12–9)	
Eq5dl	16	57.1 (25.4)	59.1 (24.2)	2.0 (16.5)	0.634	66.1 (20.4)	9.0 (13.9)	0.021*
		55(13–100)	65 (10–100)	0 (–40–30)		70 (25–100)	3 (–10–35)	

Diff: difference; SD: standard deviation; Min: minimum; Max: maximum; n: numbers; FU1: follow-Up 1; FU2: Follow- Up 2; LT: long term; BoNT: Botulinum Toxin Injection; VAS: visual analogue scale; GRT: Grasp and Release Test; COPM: Canadian Occupational Performance Measure; P: performance scale; S: satisfaction scale; ArmA: Arm Activity Measure; ArmA A: passive subscale; ArmA B: active subscale. Statistical analyses of changes in median scores were made with a Wilcoxon signed-rank test for *n* ≥ 10; *p* < 0.05 were considered significant and are presented in bold numbers, significant values after the Holm- Bonferroni method was applied are presented with *.

## DISCUSSION

The findings of the present study indicate that spasticity-corrective surgery produces beneficial gains that exceed and last beyond those achieved with BoNT. Primarily, surgery appears to suppress the symptoms of spasticity, as measured by the MAS, to a greater extent than BoNT. The mean reduction in the composite MAS score was significantly higher in the surgery group than in the BoNT group at both the peak and long-term assessments. These results are in accordance with prior studies investigating the efficacy of tendon lengthening techniques in reducing muscle hypertonia (13, 26, 27), and contribute to the existing body of knowledge in the field.

Surgery was associated with significant improvements that exceeded those achieved in the BoNT group in the MAS primary outcome and many secondary outcome measures. Specifically, surgery showed superior results in 7 of 17 secondary outcomes compared to the peak effect of BoNT, and in 11 of 17 secondary outcomes compared to the long-term effect of BoNT. These findings indicate that surgery not only reduces spasticity, but also enhances UL use. Although previous studies have shown that the effect of BoNT on active voluntary muscle function is limited (12), other studies have revealed that surgery improves active voluntary muscle function (13, 26, 28, 29), which is consistent with our findings.

The long-term effect of surgery compared with that of BoNT is anticipated to be more effective, as demonstrated in the present study. The peak effect of BoNT is transient, typically lasting for approximately 3 months. While BoNT showed significant improvements at the peak effect, these gains decreased over time, necessitating repeated injections to maintain the associated benefits. In contrast, surgical intervention requires an initial higher input from healthcare and patient but provided lasting improvements, reduced the need for continuous medical interventions, and potentially lowered associated healthcare costs. While further studies are required to determine the exact duration of these effects, prior studies have shown lasting results up to 6 years postoperatively (27).

Patient satisfaction ratings of the treatments confirmed the quantitative findings, with higher satisfaction scores in the surgical group (Mean VAS score=7.4 vs 5.9). Although both groups found their respective treatments equally demanding, the willingness of all 17 patients in the surgery group and most patients in the BoNT group to recommend the procedure to others highlighted the perceived value and acceptability of surgery and BoNT as treatment options for spasticity.

The results of the present study have significant implications for clinical practice, indicating that spasticity-corrective surgery should be considered as a treatment option for patients with UL spasticity, particularly when long-term management is the goal. Although BoNT remains a valuable tool for both immediate and short-term relief, its limitations in sustaining functional improvements necessitate the exploration of surgical options for eligible patients. Prior studies have emphasized the need for improved spasticity management (3, 7, 30, 31). Surgery is generally considered in severe cases, or as the last option for adult patients when noninvasive treatments fail (32), and is sometimes not mentioned as an option at all (33, 34). A recent review of focal spasticity management recommended surgery in 4 of 13 papers (35). The underutilization of UL surgery has been criticized by surgeons who see it as a missed opportunity (36–39). The reasons for this underutilization include a lack of knowledge about surgical options, a variety of procedures without a clear algorithm, limited outcome evidence or consensus, unfavourable past experiences, limited access to surgery, and insufficient collaboration between surgeons and rehabilitation therapists (36, 38). It is estimated that 10% of patients with spasticity could benefit from surgery (37). Some surgeons advocate considering surgery in patients with significant spasticity (36). Reports indicate that less than 1% of patients with TBI or stroke with residual spasticity undergo surgery (38). The promising results of this study could lead to increased referrals and evaluations of surgery as a treatment option for spasticity.

It is essential to note the individualized nature of spasticity management, in which treatment plans should consider patient-specific factors, including the severity of spasticity, functional and activity goals, and overall health status. One multidisciplinary approach integrating surgical interventions with comprehensive rehabilitation programs can optimize outcomes and enhance the quality of life of patients with CNS-induced spasticity.

### Limitations and future research

This study has several limitations that should be considered. Firstly, the quasi-experimental design and relatively small sample size of this study warrant cautious interpretation of our findings. Allocation based on patient preferences, while reflecting real-world scenarios, may introduce a selection bias. Future randomized controlled trials (RCT) with larger cohorts are required to confirm these results, and to further explore the comparative effectiveness of different spasticity management strategies (40). The surgical field has lagged behind other specialities in performing RCTs (41). Indeed, it has been reported that only 7% of articles published in surgical journals are RCTs (40). The reasons for the lack of RCTs on surgery include ethical issues, patient and surgeon preferences, irreversibility of surgical treatment, increased expense and follow-up time, and difficulty with randomization and blinding. In a review of meta-analyses (40), the authors concluded that the results of well-designed observational studies did not systematically overestimate the magnitude of the effects of treatment compared with those of RCTs on the same topic. Another limitation is that the outcome assessors were not blinded. Owing to the physical components of interventions, *blinding is not easily applicable in surgical studies.*

In the present study, the Holm–Bonferroni method was used for multiple statistical tests. Sample size calculations were not performed for secondary outcome measures or subgroup analyses; therefore, we may have failed to detect some important treatment effects. Therefore, the results should be interpreted with caution. Patients with varying levels of residual muscle function (different treatment regimens) and diagnoses were analysed. In the surgery group, 47% of the patients suffered from a SCI, whereas the proportion of individuals with SCI was limited to 6% in the BoNT group. In the surgery group, 29% were stratified into the HFR group compared to 35% in the BoNT group. To address the issue of differing clinical characteristics between the 2 treatments, a future experimental study using a paired design, in which different treatment regimens are analysed separately, could be of significant interest.

Overall, the findings of the present study suggest that surgical options should be included in the treatment paradigm for spasticity, tailored to the individual needs of the patient, and complemented by targeted rehabilitation programs. The high patient satisfaction ratings further validated our quantitative findings, with higher satisfaction scores and a unanimous willingness to recommend surgical treatment, demonstrating its acceptability and perceived value. The implications for clinical practice are profound, advocating for the inclusion of surgical options in spasticity management paradigms, particularly for patients seeking long-term improvements in UL function.

### Conclusion

Spasticity-corrective surgery produces beneficial gains that exceed and last beyond those achieved with BoNT in patients with disabling UL spasticity. The composite MAS scores were higher in the surgical group. Although BoNT remains a valuable tool for immediate relief, its transient nature necessitates repeated interventions, making surgery a more viable long-term solution for managing spasticity. The study’s quasi-experimental design and small sample size warrant a cautious interpretation of the results. Future studies with larger cohorts are essential to confirm these findings and further explore the comparative effectiveness of different spasticity management strategies.
